# Identifying Circular RNA and Predicting Its Regulatory Interactions by Machine Learning

**DOI:** 10.3389/fgene.2020.00655

**Published:** 2020-07-21

**Authors:** Guishan Zhang, Yiyun Deng, Qingyu Liu, Bingxu Ye, Zhiming Dai, Yaowen Chen, Xianhua Dai

**Affiliations:** ^1^School of Electronics and Information Technology, Sun Yat-sen University, Guangzhou, China; ^2^Key Laboratory of Digital Signal and Image Processing of Guangdong Provincial, College of Engineering, Shantou University, Shantou, China; ^3^School of Data and Computer Science, Sun Yat-sen University, Guangzhou, China; ^4^Guangdong Province Key Laboratory of Big Data Analysis and Processing, Sun Yat-sen University, Guangzhou, China; ^5^Southern Marine Science and Engineering Guangdong Laboratory, Zhuhai, China

**Keywords:** circular RNA, long non-coding RNA, microRNA, RNA binding protein, transcriptional regulation, machine learning

## Abstract

Circular RNA (circRNA) is a closed long non-coding RNA (lncRNA) formed by covalently closed loops through back-splicing. Emerging evidence indicates that circRNA can influence cellular physiology through various molecular mechanisms. Thus, accurate circRNA identification and prediction of its regulatory information are critical for understanding its biogenesis. Although several computational tools based on machine learning have been proposed for circRNA identification, the prediction accuracy remains to be improved. Here, first we present circLGB, a machine learning-based framework to discriminate circRNA from other lncRNAs. circLGB integrates commonly used sequence-derived features and three new features containing adenosine to inosine (A-to-I) deamination, A-to-I density and the internal ribosome entry site. circLGB categorizes circRNAs by utilizing a LightGBM classifier with feature selection. Second, we introduce circMRT, an ensemble machine learning framework to systematically predict the regulatory information for circRNA, including their interactions with microRNA, the RNA binding protein, and transcriptional regulation. Feature sets including sequence-based features, graph features, genome context, and regulatory information features were modeled in circMRT. Experiments on public and our constructed datasets show that the proposed algorithms outperform the available state-of-the-art methods. circLGB is available at http://www.circlgb.com. Source codes are available at https://github.com/Peppags/circLGB-circMRT.

## Introduction

Circular RNA (circRNA) constitutes a unique class of RNAs that is characterized by the presence of a covalently closed cyclic structure without a poly adenylated tail (Lasda and Parker, 2014). During pre-mRNA splicing, the 5′ and 3′ termini of exons can be covalently ligated to form circRNAs (Barrett et al., 2015; Wang and Wang, 2015). Owing to their circular structure and lack of free ends (Awasthi et al., 2018), circRNAs have greater stability and are more conserved across species than linear RNAs (Jeck et al., 2013). Although the functions of most circRNAs are still elusive, they have been shown to act as sponges to microRNAs (miRNAs; Hansen et al., 2013; Panda, 2018) and may potentially sponge RNA binding proteins (RBPs; Memczak et al., 2013). In addition, circRNAs can also be involved in transcriptional regulation (TR) and alternative splicing (Zhang et al., 2013; Conn et al., 2017). circRNAs may even have translation potential (Li et al., 2018). circRNAs play crucial roles in gene regulation and the development of many complex diseases. Moreover, circRNAs have a promising potential as biomarkers of diseases due to their stability and relation to diseases (Zhang et al., 2018).

Circular RNAs have some different attributes from other long non-coding RNAs (lncRNAs), such as back-splicing (Xiong et al., 2015). Unlike lncRNA, which can be effectively recognized from other little non-coding RNAs (e.g., miRNA, siRNA, and snoRNA) according to the transcript size, it is scarcely possible to distinguish circRNA from different lncRNAs based on simple features (Xiong et al., 2015). Moreover, it is hard to classify circRNAs from other lncRNAs due to the low expression levels of almost all lncRNAs. To date, several machine learning-based methods have been developed for circRNA detection. For example, PredcircRNA (Pan and Xiong, 2015) identifies circRNAs by utilizing a multiple kernel learning-based (MKL) framework. This tool incorporates diverse sequence features including basic sequence features, graph features, conservation scores as well as features of transposable element (ALU), tandem repeats, ORF length, ORF proportion, and single nucleotide polymorphism (SNP) density (ATOS) to train and test the model. Hierarchical extreme learning machine (H-ELM; Chen et al., 2018) extracts identical features and discriminates circRNAs by performing a H-ELM algorithm with feature selection. circDeep (Chaabane et al., 2019) distinguishes circRNAs by integrating a reverse complement matching descriptor, an asymmetric convolutional neural network (CNN) combined with bidirectional long short-term memory sequence descriptor and a conservation descriptor for extracting high level abstract features of a given RNA sequence. When evaluating the performance on the published dataset proposed by Pan and Xiong (2015), circDeep achieves an improvement of over 12% in terms of accuracy (ACC) compared with PredcircRNA and H-ELM (with values of 0.778 vs. 0.789). However, there is still room for improving the performance. Thus, novel computational methods and comprehensive exploration of informative sequence features affecting back-splicing are required.

Technological obstacles for understanding the regulation and functions of circRNAs occur at various levels. Take suppression strategy as an example, it usually uses loss and gain functions to annotate gene function [i.e., RNAi (Boutros and Ahringer, 2008) and CRISPR/Cas9-mediated genome editing (Shalem et al., 2015)]. However, this technique does not have adequate ability to achieve specificity or high efficacy in targeting circRNAs. Therefore, decoding the regulatory interactions of circRNAs can greatly expand the understanding of their functions. Thanks to the development of high-throughput sequencing, alongside the advance of bioinformatics technology, a great number of circRNAs loci have been discovered in human genomes. Several databases and resources are available for describing the circRNAs regulatory interactions, which can facilitate research on miRNA, RBP, and TR interacting with specific circRNAs. For instance, Circ2Traits (Ghosal et al., 2013) predicts interactions between the disease-associated miRNAs and circRNAs. CircNet (Liu et al., 2016) provides circRNA–miRNA–gene regulatory networks and tissue-specific circRNA expression profiles. CircInteractome (Dudekula et al., 2016) explores circRNAs interacting with miRNAs. Besides, it identifies RBPs binding to circRNA junctions. CIRCpedia v2 (Dong et al., 2018) provides a comprehensive circRNA annotation from over 180 RNA-seq datasets across six different species. ENCORI (Li et al., 2014) identifies the miRNA–ceRNA, miRNA–ncRNA, and protein–RNA interaction networks. TRCirc (Tang et al., 2018) provides a resource for efficient retrieval, browsing and visualization of TR information of circRNAs. The availability of these databases speeds up the exploration of circRNAs biogenesis and the function analysis.

Machine learning has made impressive advances in the area of bioinformatics such as molecular interactions prediction. The machine learning-based predictors require considerable domain expertise to design the feature extractor. For example, Muppirala et al. (2011) proposed support vector machine (SVM) and random forest (RF)-based methods to predict the RNA–RBP interactions using sequence composition. Previous studies suggested that incorporating informative features can boost the predictive power (Ahmed et al., 2009, 2013; Wang L. et al., 2019). For instance, Ahmed et al. (2009) proposed SVM-based methods to predict guide strand of miRNAs and human Dicer cleavage sites (Ahmed et al., 2013). In their work, they found adding secondary structure information contributes to the improvement of ACC compared with considering sequence only. Owing to the non-coding nature of circRNA, the relationship between structure and function in it is stronger than in linear RNAs. There is increasing evidence that RNA secondary structure promotes exon skipping RNA circularization (Pervouchine, 2019) and alternative splicing (Buratti and Baralle, 2004). Besides, a quantitative characterization of the relationship between primary sequence and structure of circRNAs contributes to our understanding of how their function emerges. Inspired by this, incorporating secondary structure features may achieve better performance than considering primary sequence for circRNAs regulatory interactions prediction. Recently, machine learning-based identification of circRNAs coordinated regulatory interaction has been gradually applied in the bioinformatics field. For example, CircRNAs Interact with Proteins (CRIP) integrates CNN and a recurrent neural network to predict circRNA–RBP binding sites (Zhang et al., 2019). Wang Z. et al. (2019) proposed a multiple CNNs-based method to identify cancer-specific circRNA–RBP binding sites considering only nucleotide sequences. Ju et al. (2019) applied a hybrid LSTM-CNN-CRF (a long short-term memory network, CNN network and a conditional random field) model to identify RBP-binding sites on circRNAs (Ju et al., 2019). Lei and Fang (2019) proposed GBDTCDA, a gradient boosting decision tree (GBDT) regression model with multiple biological data to predict circRNA-disease associations (Lei and Fang, 2019). To the best of our knowledge, no machine learning-based tool has been proposed to systematically predict the regulatory information of circRNAs, including their interactions with miRNA, RBP, and TR.

In this study, we introduce two machine learning-based methods, circLGB and circMRT to combine both sequence and structure information, to identify circRNAs from other lncRNAs and to predict their regulatory interactions, respectively. circLGB extracts the commonly used features and three new features including adenosine to inosine (A-to-I) deamination, A-to-I density as well as internal ribosome entry site (IRES), and in turn, distinguishes circRNA by utilizing a LightGBM classifier. We propose a two-step feature optimization strategy to select the most discriminative features. circLGB achieves superior performance on the public and our datasets compared to the state-of-the-art methods. circMRT integrates sequence-based features, graph features, genome context and regulatory information for predicting circRNA interacting with miRNA, RBP, and TR. We first propose three classifiers to predict circRNA–miRNA, circRNA–RBP and circRNA–TR interactions, respectively. Each classifier extracts the abovementioned sequence features and predicts the regulatory interaction by applying an ensemble machine learning algorithm with optimal features. Then, the outputs of all three classifiers are fused by a union operator to predict the coordinated regulatory interaction of the candidate circRNA. As far as we know, circMRT is currently a comprehensive computational platform that predicts the regulatory information of circRNA using machine learning.

## Materials and Methods

### Data Collection and Pre-processing

#### circlncRNA Datasets

We downloaded the human circRNAs from the circBase (Glazar et al., 2014) database. Taking circRNA isoforms into consideration and removing the transcripts which were shorter than 200 nt, we obtained 79,987 positive samples. Besides, we also downloaded the annotated human lncRNAs from LNCipedia (Volders et al., 2013). This database provides basic transcript information, gene structure and several statistics (e.g., miRNA binding sites and secondary structure) for each transcript. After excluding the overlapped circRNAs in circBase and deepBase (Zheng et al., 2016), we obtained 127,432 lncRNAs transcripts. We randomly selected 21,882 circRNAs and the same number of lncRNAs to construct our circlncRNA dataset. The determination of the sample size is given in Supplementary Material (Supplementary Figure S1).

#### CIRCdeep Dataset

We used a dataset available in Chaabane et al. (2019) (hereafter referred to as CIRCdeep). This dataset contains 32,914 human circRNAs and 19,683 lncRNAs. circRNAs were downloaded from the circRNADb (Chen et al., 2016) database. Transcripts shorter than 200 nt were removed. Negative data was collected from the GENCODE (Harrow et al., 2012) database. The annotated lncRNAs in GENCODE have three validation levels for RNA annotation, namely validation, manual annotation, and automated annotation. Only validated or manually annotated transcripts were chosen. CIRCdeep dataset can be downloaded at https://github.com/UofLBioinformatics/circDeep.

#### circMI, circRBP, circTR Datasets and the Independent Test Set

circRNA–miRNA and circRNA–RBP interactions were downloaded from the ENCORI database^1^. Additionally, circRNA–TR interactions were extracted from the TRCirc database^2^. After removing the duplicates and getting the full-length sequence and basic sequence information from circBase database, we built datasets circMI, circRBP, and circTR for training the classifiers of circRNA–miRNA, –RBP and –TR, respectively. To be specific, we randomly selected 1046 full-length circRNAs interacting with miRNAs to construct the positive data of circMI dataset. We collected 1036 and 2172 entire circRNAs which have interactions with RBPs and TRs, being used as positive data of circRBP and circTR datasets, respectively. Note that, there is no overlap among these three positive samples. We randomly selected 1046 circRNAs interacting with TRs as negative data for circMI dataset. Analogously, 1036 circRNAs interacting with TRs were derived to be used as negative samples for circRBP dataset. The 2172 circRNAs interacting with miRNAs or RBPs were chosen to construct the negative data of circTR dataset. In addition, we used 140 samples including 29 circRNA–miRNA interactions, 50 circRNA–RBP interactions, 40 circRNA–TR interactions, and 21 miRNA–circRNA-RBP interactions as an independent test set. This test set does not overlap the former datasets. More details can be found in Table 1.

**TABLE 1:** Summary of circMI, circRBP, circTR datasets and the independent test set.

Model	Dataset	Positive data	Negative data
circRNA-miRNA	circMI	1046 circRNAs interacting with miRNAs	1046 circRNAs interacting with TRs
circRNA-RBP	circRBP	1036 circRNAs interacting with RBPs	1036 circRNAs interacting with TRs
circRNA-TR	circTR	2172 circRNAs interacting with TRs	2172 circRNAs interacting with miRNAs or RBPs
circMRT	Independent test set	–	–

### Feature Extraction

Feature extraction has great influence on the predictive performance. Note that, features related to RNA circularization and circRNA regulatory information may be different. So, we separately extracted the features for circLGB and circMRT models. 188 sequence-derived features including 70 sequence composition features, 101 graph features, 12 conservation scores, and 5 ATOS features (Pan and Xiong, 2015; Chen et al., 2018) were used for circRNAs detection. Based on these features, we added three features including A-to-I, A-to-I density and IRES to train our circLGB. We extracted a 182-dimensional vector to train our circMRT for circRNAs regulatory interactions prediction. These features were divided into four groups: sequence-based features, graph features, genome context and regulatory information. The value of each feature was normalized to the interval from 0 to 1. More details were summarized in Supplementary Tables S1, S2.

#### Features of circLGB for Classifying circRNA From Other lncRNAs

##### Group 1: Basic sequence features

The basic sequence features were extracted using the same processing scheme described in Pan and Xiong (2015). These features contain a wide range of possible explanatory attributes from 64 trinucleotide frequencies and other sequence component composition features, e.g., sequence length, GC content, frequencies of AG, GT, AGGT, and GTAG. GT/AG signal has an impact on forming the circRNAs, such as back-splicing and exon-junction (Kitamura-Abe et al., 2004). A detailed description can be referred to Pan and Xiong (2015).

##### Group 2: Graph feature

RNA structure plays an important role in gene splicing, which has an influence on back-splicing (Ding et al., 2014). Secondary structures play important role in identifying of the hypothetical interacting sites of circRNAs (Cuesta and Manrubia, 2017). In RNA graph, the nodes are nucleotides while edges represent backbone connection or bond relations between the nucleotides (Maticzka et al., 2014). RNA graph features reflect the relationships between nucleotides and represent the relations of the abstract structure annotations predicted from RNA shapes (Steffen et al., 2006). GraphProt is a machine learning-based framework considering both sequence and full secondary structure information that can find RBP sequence and structure-binding preferences from the high-throughput data (Maticzka et al., 2014). In this work, we applied GraphProt to calculate RNA secondary structures. In addition, it was adopted in previous studies (Pan and Xiong, 2015; Chen et al., 2018; Pan et al., 2018; Ilik et al., 2020). We initially extracted a 32,768-dimensional RNA graph feature vector for the candidate transcript using GraphProt 1.0.1. To improve the feature representation ability, Pan et al. employed RF to rank the extracted features by their importance scores and chose the top 101 features (Pan and Xiong, 2015). For fair comparison, we used these 101 features for analysis. The RF importance ranking list of the selected features can be downloaded from https://github.com/xypan1232/PredcircRNA/blob/master/features/all_fea_ranking.

##### Group 3: Conservation scores

Previous studies showed that circRNAs are significantly enriched with conserved nucleotides (Memczak et al., 2013). On the contrary, lncRNAs have a low level of sequence conservation compared with other functional transcripts (Marques and Ponting, 2009). Thus, conservation scores may help to discriminate circRNAs from lncRNAs. These scores were extracted by downloading the placent_phylop46way^3^ from the UCSC database (Karolchik et al., 2003). We calculated the mean, maximum, and variance of conservation scores from per base phyloP conservation score for each transcript (Lowe et al., 2011). Furthermore, the frequencies of bases with conservation scores greater than 0.3, 0.6, 0.9 and smaller than 0.9 were also calculated.

##### Group 4: ALU and tandem repeat, ORF, SNP, IRES, A-to-I, and A-to-I density

ALU repeats contribute to RNA circularization by making the splice sites recognize each other (Liang and Wilusz, 2014). We downloaded the annotated ALU repeat sites from UCSC and calculated the number of ALU repeats for each transcript. Tandem duplications within a gene have a great impact on back-splicing (Ulitsky et al., 2011). Tandem repeats were detected by employing Tandem Repeat Finder (Benson, 1999). We computed the frequency of tandem repeats. The open reading frame (ORF) length information was extracted by using txCdsPredict from UCSC. The longest ORF and ORF propensity (ORF prop) defined by the length of an ORF divided by the total length of the transcript were calculated. Single nucleotide polymorphism data with coordinates in the genome was downloaded from the 1000 Genomes Project (Kuehn, 2008). Single nucleotide polymorphism density was computed for each transcript. A previous study suggested that A-to-I editing events occur frequently at intronic positions that were proximal to the splice sites of circularized exons (Ivanov et al., 2015). The annotated data of A-to-I was downloaded from the RADAR (Ramaswami and Li, 2014) dataset. A-to-I density was defined by the number of A-to-I divided by the sequence length for each transcript. Another work demonstrated that IRES provides the information of peptides or proteins from circRNA (Abe et al., 2015), implying that this feature has discriminative power for circRNA detection. IRES information of the given RNA sequence was extracted by IRESfinder (Zhao et al., 2018).

#### Features of circMRT for Predicting circRNA Regulatory Interactions

##### Group 1: Sequence-based features

The sequence features consist of 70 sequence composition features and one repeat feature. Note that these features were generated in the same way in section “Features of circLGB for Classifying circRNA From Other lncRNAs.”

##### Group 2: Graph features

The 101-dimensional graph features were generated identically to the way described in section “Features of circLGB for Classifying circRNA From Other lncRNAs.”

##### Group 3: Genome context features

We calculated the mean and standard deviation of conservation scores for each transcript. ALU, SNP density and A-to-I features were generated identically to the way described in section “Features of circLGB for Classifying circRNA From Other lncRNAs.” A previous study showed that circRNA sequences are enriched for back-splice junctions (Jeck et al., 2013). Moreover, CIRI (Gao et al., 2015) and find_circ (Memczak et al., 2013) characterized circRNA by calculating the circular junctions. We derived the one-dimensional back-splice junction feature from the TRCirc database. It is a general phenomenon that circRNAs compete with other RNAs for binding miRNAs. For example, ciRS-7 contains over 70 selective conserved miRNA target sites (Hansen et al., 2013). We integrated the one-dimensional miRNA binding sites as one feature.

##### Group 4: Regulatory information features

Transcriptional regulation involves in a complex and meticulous pattern of activities that incorporates with transcription factors (TFs; Rowell et al., 2014). A recent study indicated that TFs can selectively promote the expression of circular Cul2 rather than the host gene (Meng et al., 2018). circRNAs are regulated by TFs and other correlative information, such as H3K27ac signals. Yang et al. found N^6^-methyladenosine boosts the efficient initiation of protein translation from circRNAs in human cells (Yang et al., 2017). We obtained the one-dimensional of TF feature vector, methylation feature vector, H3K27ac feature vector from TRCirc for each sequence, thereby leading to a 3-dimensional vector.

### Model Training and Optimization

#### LightGBM

Gradient boosting decision tree (Friedman, 2001) is an iterative decision tree algorithm with various effective implementations such as XGBoost (Chen and Guestrin, 2016). However, the efficiency and scalability are still ungratified when feature dimension is high and data size is large (Ke et al., 2017). Recently, LightGBM (Ke et al., 2017) has been proposed to address this issue, which can effectively solve the time-consuming problem of conventional GBDT while retaining high classification ACC. LightGBM possesses two novel techniques: gradient-based one-side sampling (GOSS) and exclusive feature bundling (EFB). Gradient-based one-side sampling excludes a significant proportion of data instances with small gradients and uses the remaining to estimate the information gain. Hence, this technique can effectively reduce the number of data at the time of calculation and further improve the efficiency. Exclusive feature bundling bundles mutually exclusive features to reduce the number of features. Features with larger gradients contribute more to the information gain and are thus more important for classification. Compared with GBDT, LightGBM speeds up the training process significantly because the number of bundled features will be much smaller than those of the original features. The speed of model training in LightGBM is 20 times faster than GBDT under the premise of achieving almost the same ACC (Ke et al., 2017). We employed the LightGBM algorithm using the lightgbm package in Python^4^.

#### Support Vector Machine

Support vector machine is one of the most widely used machine learning algorithms for classification problems (Noble, 2006). The main idea of SVM is based on kernel functions that map the input data into a high dimensional space. Support vector machine aims to search the hyperplane to maximize the margin between two support vectors. In this study, SVM with the “linear” kernel was implemented using the Scikit-learn library in Python. We optimized the parameter cost C from the choice of (1.0, 1.1, 1.2, 1.3, 1.4) by grid search. After optimization, the parameter of C was set as 1.0.

#### Random Forest

Random forest (Liaw and Wiener, 2002) is an ensemble learning method for regression and classification which involves multiple decision trees. Random forest assumes that there are P samples with Q features in the original training set, and it selects P samples from the training data by bootstrapping and randomly selects q features (q≪Q) to train a decision tree. By repeating the step above, numerous decision trees are trained, and their outputs are integrated in the ensemble model to make a final prediction. We trained the RF with 20 decision trees using Scikit-learn.

#### Stochastic Gradient Descent

Stochastic gradient descent (SGD; Friedman, 2001) is an effective method for solving large scale supervised machine learning problems. It generally confers a significant decrease in training time without sacrificing ACC. In particular, SGD with early stopping at a fixed number of interactions approximately halves the training time. In this work, SGD was applied using Scikit-learn.

#### Gaussian Naive Bayes

A Naive Bayes (NB) classifier calculates the probability of a given example belonging to a certain class. When the likelihood of the features is assumed to be Gaussian, the NB classifier is called Gaussian naive Bayes (GNB; John and Langley, 2013). Gaussian naive Bayes supposes that features are independent from each other. Gaussian naive Bayes is simpler and faster than other sophisticated methods. Thus, it is usually used for prediction problems in bioinformatics (Murakami and Mizuguchi, 2010). Here, GNB was also implemented using Scikit-learn.

### circLGB

**FIGURE 1:**
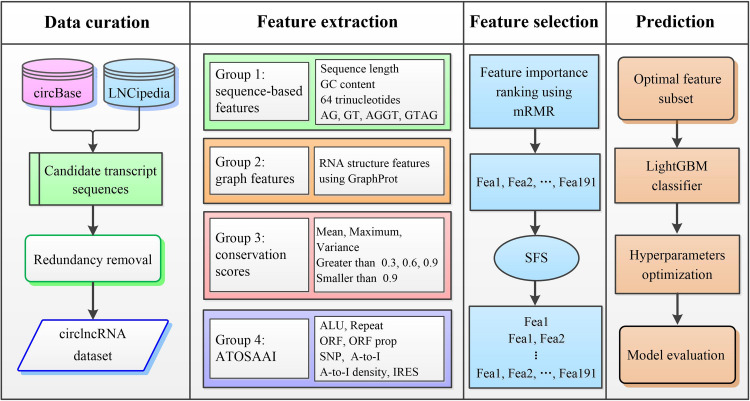
Overview of the proposed circLGB for circRNA identification that involved the following steps: **(i)** construction of circlncRNA dataset; **(ii)** extraction of sequence-derived features including sequence composition, graph features, conservation scores and ATOSAAI for training and testing the circLGB model; **(iii)** ranking the features using mRMR algorithm according their importance and generation of the optimal feature subset using SFS; and **(iv)** construction of the final prediction by applying LightGBM classifier that separates the input into circRNAs and lncRNAs. ALU, transposable element; ATOSAAI, ATOS, A-to-I, A-to-I density and IRES; ORF, open reading frame; SNP, single nucleotide polymorphism; A-to-I, adenosine to inosine; mRMR, minimal redundancy and maximal relevance; SFS, sequential forward search. These abbreviations also apply to Figures 2, 3.

We proposed a machine-learning framework called circLGB to classify circRNA from lncRNAs. As shown in Figure 1, the major procedures of circLGB can be summarized as below: (i) The collected human circRNAs and lncRNAs transcripts are combined to construct the circlncRNA dataset. (ii) Four groups of sequence-derived features are extracted from various toolkits and databases. (iii) minimum redundancy-maximum relevance (mRMR; Ding and Peng, 2005) feature selection framework is applied to rank the extracted features according their importance scores. Then, sequential forward search (SFS) is utilized to determine the optimal feature subset which yields the best Matthews correlation coefficient (MCC). Supplementary Table S3 summarizes the feature importance scores on the circlncRNA dataset. (iv) The resulting feature vector is fed into the LightGBM classifier for circRNA identification. Finally, performance metrics are calculated for model evaluation.

### circMRT

**FIGURE 2:**
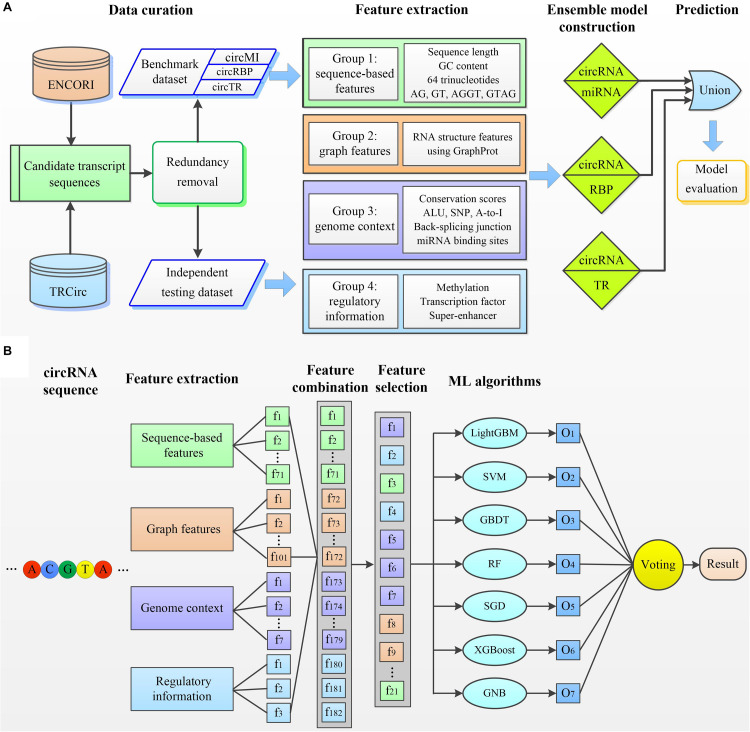
The overall framework of circMRT. **(A)** An outline of the overall flowchart of circMRT. The development of circMRT involved four major steps: **(i)** data collection and preprocessing, **(ii)** feature extraction, **(iii)** ensemble model construction, and **(iv)** model prediction and performance assessment. **(B)** An illustration of the detailed procedures for constructing the circRNA-miRNA classifier. (i) Four groups of features including sequence composition, graph features, genome context and regulatory information are extracted for each candidate circRNA. (ii) The optimal feature set is selected by applying the proposed two-step feature selection strategy. **(iii)** Based on the optimal feature set, we train the prediction models using seven machine learning-based algorithms. **(iv)** Three individual model’s outputs are integrated by using majority voting algorithm.

We next developed circMRT to predict the regulatory information for circRNAs, including their interactions with miRNA, RBP, and TR. Note that, one interaction may exist simultaneously for a given circRNA. We first developed three binary classifiers to explore whether the given circRNA has associations with miRNA, RBP, and TR, respectively. Then, the outputs of these classifiers were fused to make a final prediction. The circMRT methodology (Figure 2) consists of four major steps: (i) Datasets circMI, circRBP and circTR are constructed to train the circRNA–miRNA, circRNA–RBP and circRNA–TR classifiers, respectively. Besides, independent test set is generated to evaluate the generalization of circMRT. (ii) The candidate circRNA sequence is input for feature encoding by extracting four types of features. (iii) The extracted features are fed into the abovementioned classifiers for training and testing. Each classifier is trained on its own optimal features selected by applying the proposed feature optimization strategy. (iv) The independent test set is respectively fed into three well-trained classifiers for prediction. Finally, the outputs are fused by a union operator to predict the regulatory interactions for a given circRNA.

### Feature Selection

We utilized a two-step feature selection strategy to improve the feature representation ability. We first used mRMR to achieve the ranked feature list according to the importance scores of the learned features. Features with higher scores were more predictive. Second, SFS was applied to investigate the optimal combination of features that can yield the best performance. We ranked the features in a descending order from the mRMR features list. Subsequently, incremental feature selection approach was employed to select the optimal top-k features. We added the features from the ranked feature list one by one and trained the proposed model. The feature subset with the relative higher values of MCC was regarded as the most discriminative features. It is worth noting that we here used the MCC since it is a balanced measurement, even if the sizes of positive and negative samples are imbalanced. Therefore, the MCC is a better indicator to assess the performance of the models.

### Hyperparameters Optimization

cricLGB and circMRT were implemented using Python 2.7. All experiments were carried out on a desktop computer with Intel (R) Core (TM) i7-7800X CPU @ 3.50GHz, Ubuntu 16.04.5 LTS and 16 GB RAM. To ensure the ACC and robustness of the proposed algorithms, we employed the grid-search parameter adjustment to achieve the optimal parameters. Specifically, we used grid-search to tune six parameters including learning rate, number of leaves, feature fraction, bagging fraction, reg_alpha, and reg_lambda for each dataset. The Grid search range of each parameter was as below: learning rate from the choice (0.01, 0.02, 0.03, 0.04, 0.05, 0.06, 0.07, 0.08, 0.09, 0.1, 0.2), number of leaves from the choice (20, 25, 30, 35, 40, 45, 50), feature fraction from the choice (0.5, 0.6, 0.7, 0.8, 0.9), bagging fraction from the choice (0.5, 0.6, 0.7, 0.8, 0.9), reg_alpha from the choice (0.001, 0.01, 0.03, 0.05), and reg_lambda from the choice of (0.001, 0.01, 0.03, 0.05). The proposed methods are binary classification problems, we used “binary” of “objective” and “auc” of ‘metric’ with 100 times iteration and “stopping patience” of 10.

Considering that the grid-search for all the parameters requires a large computation cost, we adjusted the above parameters in batches to maximize the value of AUC under 5-fold cross-validation. We took the optimal hyperparameters for the model once the performance does not improve. The tuned optimal parameters were regarded as the input parameters to tune the next parameters. We first tuned the parameters of learning rate and number of leaves. Then, we adjusted the feature fraction and bagging fraction. Next, we tuned the regularization parameter including alpha and lambda. The combination of the optimal parameters for circLGB from the learning rate of 0.1 were, number of leaves of 60, feature faction of 0.5, bagging fraction of 0.6, reg_alpha of 0.01 and reg_lambda of 0.001. The determination of the optimal parameters of circRNA–miRNA, circRNA–RBP and circRNA-TR classifiers were as follows: learning rate of (0.1, 0.1, 0.1), number of leaves of (20, 40, 35), feature faction of (0.6, 0.6, 0.6), bagging fraction of (0.6, 0.6, 0.5), reg_alpha of (0.01, 0.001, 0.01), and reg_lambda of (0.03, 0.001, 0.01).

### Performance Evaluation

To evaluate the performance of our models and to compare with existing state-of-the-art methods, sensitivity (SE), specificity (SP), precision (PRE), F1 score (F1), ACC, and MCC were calculated. These indicators are widely used to measure the quality of binary classification defined as follows:

(1) $$\text{SE}=\frac{T\,P}{T\,P+F\,N}$$

(2) $$\,\text{SP}=\frac{T\,N}{T\,P+F\,P}$$

(3) $$\text{PRE}=\frac{T\,P}{T\,P+F\,P}$$

(4) $${\text{F}}_{1}=2\times \frac{S\,N\times P\,R\,E}{S\,N+F\,R\,E}$$

(5) $$\text{ACC}=\frac{T\,P+T\,N}{T\,P+F\,P+T\,N+F\,N}$$

(6) $$\text{MCC}=\frac{\left(T\,P\times T\,N\right)-\left(F\,N\times F\,P\right)}{\sqrt{\left(T\,P+F\,N\right)\times \left(T\,N+F\,P\right)\times \left(T\,P+F\,P\right)\times \left(T\,N+F\,N\right)}}$$

where TP, TN, FP, and FN represent the numbers of true positives, true negatives, false positives, and false negatives, respectively. Receiver Operating Characteristic (ROC) curves were employed to visualize the performance between different methods together with the area under ROC curve (AUC).

## Results

### circLGB for circRNA Identification

#### The Effect of Three New Sequence-Derived Features

**FIGURE 3:**
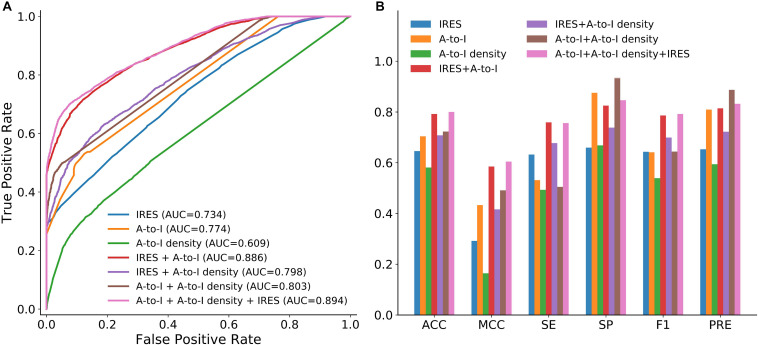
Performance of circLGB for circRNA identification on the circlncRNA dataset by various combinations of sequence-derived features including IRES, A-to-I and A-to-I density. Panel **(A)** shows comparison of ROC curves and panel **(B)** shows comparison of ACC, MCC, SE, SP, F1,3] and PRE.

We first examined whether A-to-I, A-to-I density or IRES could be used as effective features for circRNA identification. To this end, we trained circLGB with these features on the circlncRNA dataset under 10-time 5-fold cross-validation. As shown in Figure 3, two observations can be made: (i) circLGB trained using IRES achieved the highest SE value of 0.632. (ii) circLGB trained with A-to-I achieved more optimal performance than those using A-to-I density or IRES. These results indicated that no single feature contains enough useful patterns and characteristics for classifying circRNAs.

**FIGURE 4:**
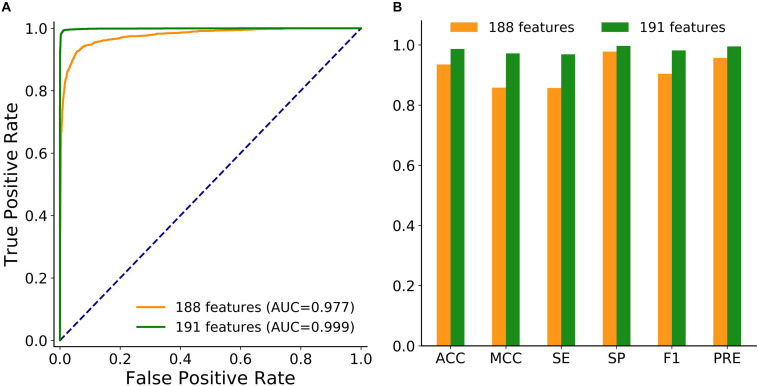
**(A)** ROC curves and **(B)** histograms showing the performance of circLGB by extracting 188 and 191 sequence-derived features on the circlncRNA dataset under 10-time 5-fold cross-validation. The performance comparison in terms of ACC, MCC, SE, SP, F1, and PRE.

To achieve better performance, the combination of these three new features was modeled in circLGB. As depicted in Figure 3, IRES combined with A-to-I outperformed any other combinations of two features, reaching AUC value of 0.886. Interestingly, though A-to-I density alone showed relatively poor performance, it gained great progress by incorporating with A-to-I or IRES, reaching AUC values of 0.803 and 0.798, respectively. circLGB achieved an overall AUC of 0.894 using these three features. So, we added them with commonly used features to train our model. As expected, circLGB trained using 191 features, achieved better performance than that on 188 features, reaching AUC values of 0.999 and 0.977, respectively (Figure 4A). Similar results on other evaluation metrics can be found in Figure 4B. Together, the addition of three new features can boost the prediction ability of circLGB.

#### Feature Importance Analysis for circLGB

**FIGURE 5:**
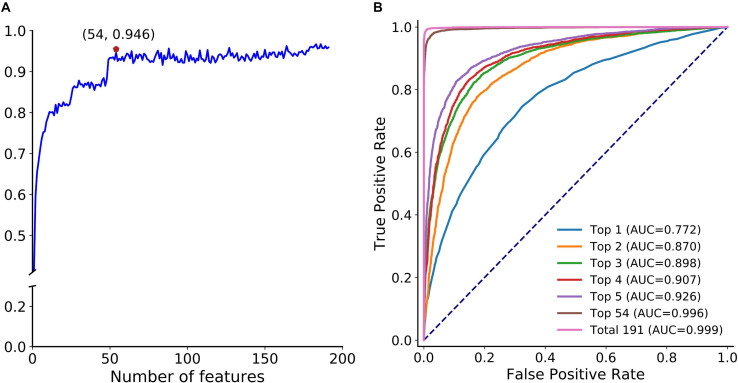
Feature importance analyses. **(A)** SFS curve of MCC with increasing number of ranked features. The features are selected by mRMR feature importance list in descending order. X-axis represents the number of selected features. The maximum MCC (0.946) obtained by integrating the top 54 features on the curve is marked by a red pentagon. This notation also applies to Figure 7A. **(B)** ROC curves of circLGB for discriminating circRNAs and lncRNAs by using the top 1 to top 5, top 54 and total 191 features.

Next, we adopted the proposed optimization strategy to enhance the feature representation ability. Figure 5A depicts the SFS curve of MCC of circLGB on the circlncRNA dataset by adding features one by one from the ranked feature list (Supplementary Table S3). Apparently, it increased quickly as the features were integrated. The MCC reached a relatively high value of 0.946 when adding the top 54 features. However, the performance fluctuated when incorporating more features. This implied that the improvement of the low-ranked features is not obvious, and they even lead to a decline of the performance. Moreover, we compared the performance of circLGB using the top 1 to top 5, top 54 and all features under 10-time 5-fold cross-validation. Obviously, the performance of circLGB trained on the selected feature sets improved when gradually adding the top ranked features (Figure 5B). The predictive results using the optimal features showed comparable performance with those using 191 features, reaching ROC values of 0.996 and 0.999, respectively. Therefore, these 54 features were regarded as the optimal features.

Supplementary Figure S2 illustrates the feature importance distribution of the optimal features based on the importance scores. There were 30 graph features, 10 sequence-based features, 9 conservation scores, and 5 ATOSAAI features (ATOS, A-to-I, A-to-I density and IRES) amongst them. This result was consistent with a recent study that shows that graph features are the most predictive features for circRNA detection (Chen et al., 2018). We noted that A-to-I density, A-to-I, and IRES features, respectively, were ranked in the 3rd, 26th, and 49th place, which verified their superior ability in identifying circRNA.

#### Comparison With Learning-Based Methods

**FIGURE 6:**
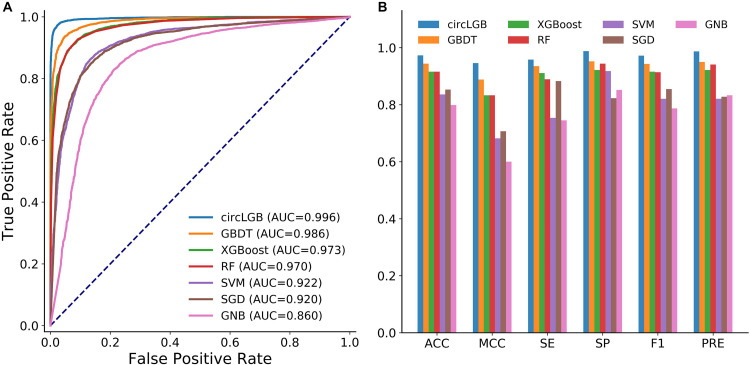
**(A)** ROC curves and **(B)** histograms of evaluation metrics show the superior performance of circLGB over GBDT, XGBoost, RF, SVM, SGD, and GNB for circRNA identification on the circlncRNA dataset under 10-time 5-fold cross-validation. Evaluation metrics including ACC, MCC, SE, SP, F1, and PRE. GBDT, gradient boosting decision tree; RF, random forest; SVM, support vector machine; SGD, stochastic gradient descent; GNB, Gaussian naive Bayes.

We compared the performance of circLGB with six machine learning algorithms including GBDT, XGBoost, RF, SVM, SGD, and GNB on the circlncRNA dataset using the optimal features under 10-time 5-fold cross-validation. All the machine learning methods were run under their optimal parameters for fair comparisons. As shown in Figure 6A, of the seven algorithms tested here, circLGB was the most predictive, with ROC of 0.996. Moreover, circLGB outperformed others with remarkable ACC, MCC, SE, SP, F1, and PRE values of 0.973, 0.946, 0.958, 0.988, 0.972, and 0.987, respectively (Figure 6B). Furthermore, we compared circLGB with two state-of-the-art predictors (e.g., circDeep and PredcircRNA) on the CIRCdeep dataset. For fair comparison, we randomly separated the dataset into a training dataset, a validation dataset, and an independent testing set with 75, 10, and 15%, respectively. Overall, circLGB achieved the most powerful predictive ability, with ACC, MCC, and F1 values of 0.998, 0.995, and 0.998, respectively (Table 2).

**TABLE 2:** Performance evaluation of our circLGB and other two learning-based algorithms on the CIRCdeep dataset.

Model	ACC	MCC	F1	References
circLGB	**0.998**	**0.995**	**0.998**	**–**
circDeep	0.942	0.883	0.940	(Chaabane et al., 2019)
PredcircRNA	0.806	0.611	0.811	(Pan and Xiong, 2015)

*The best performance across different evaluation metrics is highlighted in bold for clarification. These highlights also apply to Tables 3, 4.*

### circMRT for Predicting circRNA Regulatory Interactions

#### Feature Importance Analysis for circMRT

We first compared the performance of the proposed classifiers. As indicated in Table 3, sequence-based features were more important than other groups of features for each classifier. The regulation information features had strong discriminating power for predicting circRNA–TR interactions. Supplementary Tables S4–S6 present the ranked feature list of circRNA–miRNA, circRNA–RBP, and circRNA–TR classifiers on datasets circMI, circRBP, and circTR, respectively. Some interesting conclusions can be drawn: (i) The predicted results of circRNA-miRNA interactions were strongly influenced by ALU and miRNA. (ii) Junction and repeat features contributed most for the circRNA–RBP classifier. (iii) Junction and methylation were the most predictive for the circRNA–TR classifier. (iv) Conservation scores ranked in the top seven features of all three classifiers. Therefore, conservation information was very predictive to distinguish circRNA regulatory interactions.

**TABLE 3:** Performance of three ensemble machine learning-based classifiers for circRNA regulatory interactions prediction based on different groups of sequence-derived features.

Features	ROC	SE	SP	ACC	MCC	F1	PRE
**(A) circRNA-miRNA classifier**							
Sequence-based	**0.995**	**1.000**	**0.991**	**0.995**	**0.990**	**0.995**	**0.990**
Graph features	0.958	0.980	0.937	0.957	0.915	0.956	0.933
Genome context	0.884	0.876	0.892	0.883	0.766	0.889	0.904
Regulation information	0.952	0.922	0.981	0.952	0.906	0.950	0.979
**(B) circRNA-RBP classifier**							
Sequence-based	**0.991**	**0.986**	**0.995**	**0.990**	**0.981**	**0.991**	**0.995**
Graph features	0.969	0.954	0.985	0.969	0.938	0.970	0.986
Genome context	0.895	0.833	0.956	0.894	0.794	0.888	0.951
Regulation information	0.955	0.925	0.985	0.954	0.910	0.954	0.985
**(C) circRNA-TR classifier**							
Sequence-based	**0.993**	**0.989**	**0.998**	**0.993**	**0.986**	**0.993**	**0.998**
Graph features	0.961	0.935	0.987	0.962	0.925	0.959	0.985
Genome context	0.891	0.954	0.829	0.891	0.788	0.896	0.846
Regulation information	0.971	0.978	0.965	0.971	0.943	0.970	0.962

**FIGURE 7:**
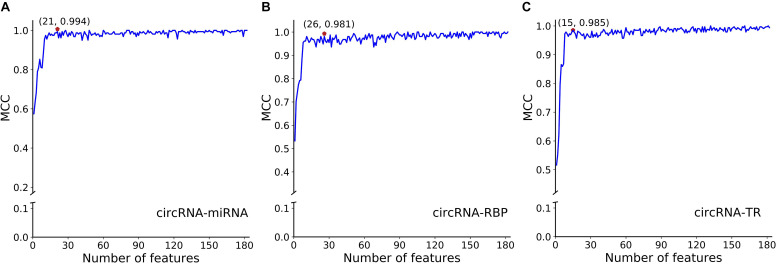
SFS curves of MCC with increasing number of selected features for classifiers of panel **(A)** circRNA-miRNA, **(B)** circRNA-RBP, and **(C)** circRNA-TR, respectively. The features are selected by their estimated feature importance in descending order. We choose the top 21, top 26, and top 15 features for the above three classifiers, with MCC values of 0.994, 0.981, and 0.985, respectively.

To avoid overfitting, we performed the proposed feature optimization strategy to obtain the representative features for each classifier. Figure 7 depicts the MCC curves of these classifiers by gradually integrating features from the ranked feature list. It can be observed that the maximum MCC values of circRNA–miRNA, circRNA–RBP, and circRNA–TR classifiers were 0.994, 0.981, and 0.985 (Table 4) when the top 21, 26, and 15 features from their own ranked feature list were used. Therefore, circRNA associated with miRNA, RBP, and TR were predicted using the proposed classifiers with their own optimal features.

**TABLE 4:** Performance evaluation of three binary classifiers on datasets circMI, circRBP, and circTR, respectively.

Classifier	Dataset	ROC	SE	SP	ACC	MCC	F1	PRE
circRNA-miRNA	circMI	0.981	0.986	0.976	0.981	**0.994**	0.981	0.977
circRNA-RBP	circRBP	0.990	**0.991**	0.990	0.990	0.981	0.991	0.991
circRNA-TR	circTR	**0.992**	0.988	**0.997**	**0.992**	0.985	**0.992**	**0.997**

#### Performance Evaluation of circMRT on the Independent Test Set

**FIGURE 8:**
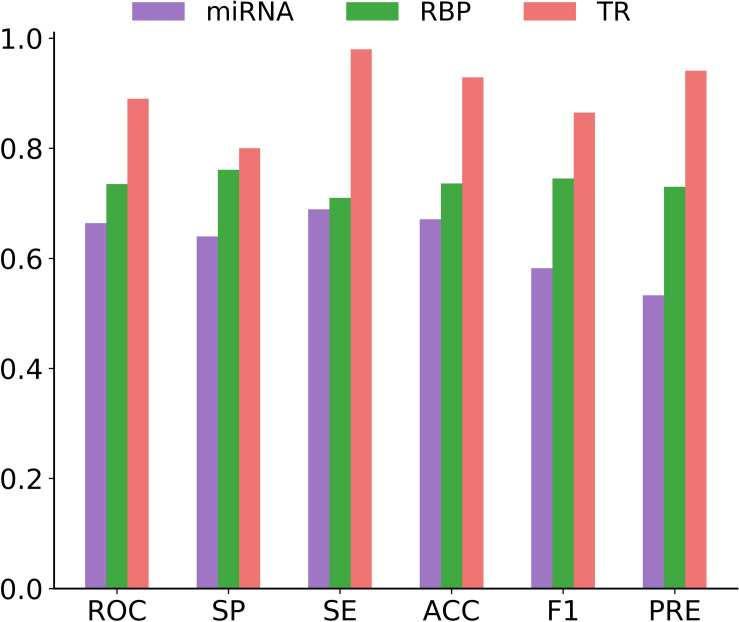
Histograms showing the performance of circRNA-miRNA, circRNA-RBP and circRNA-TR classifiers in terms of ROC, SP, SE, ACC, F1, and PRE on the independent testing dataset.

In this section, we focused on measuring the generalizability of circMRT for unseen data. For this purpose, we evaluated the performance of circMRT on the constructed independent test set. This dataset was split into 60, 20, and 20% classes, subsequently being used as the training set, validation set, and testing set, respectively. As depicted in Figure 8, circRNA-TR classifier exhibited the best predictive power, with the maximal ROC and ACC values of 0.890 and 0.929. The circRNA-RBP classifier was the second most predictive, with ROC and ACC of 0.735 and 0.736, respectively. The circRNA-miRNA classifier also performed well, but with relatively lower ROC and ACC.

**FIGURE 9:**
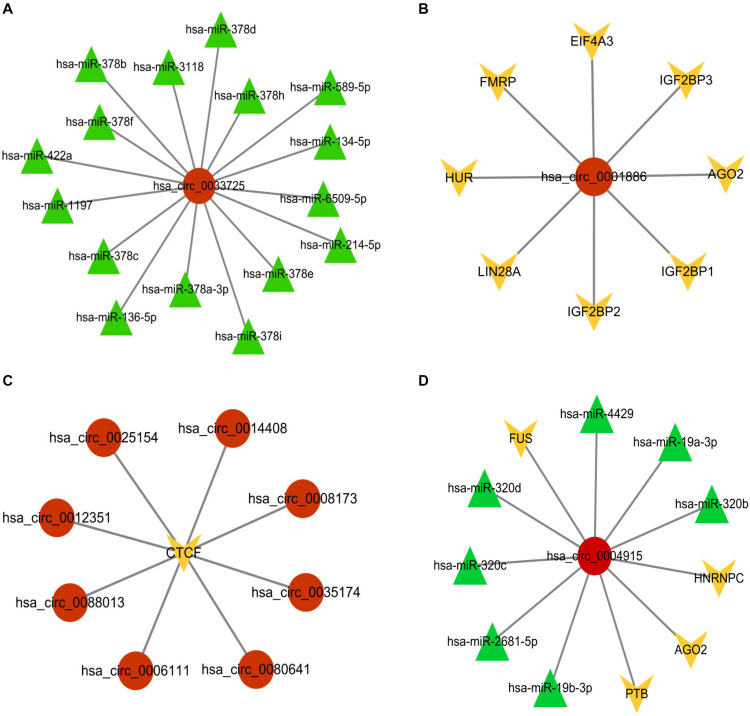
Visualization of the circRNA-associated interactions according to databases ENCORI, TRCirc and Interactome. **(A)** Visualization of the has_circ_0033725 associated with miRNAs interactions. **(B)** Visualization of the has_circ_001886 associated with RBPs interactions. **(C)** Visualization of has_circ_0006111, has_circ_0008173, has_circ_0012351, has_circ_0014408, has_circ_0025154, has_circ_0035174, has_circ_0080641, and has_circ_0088103 associated with TR interactions. **(D)** Visualization of has_circ_0004915 associated with miRNAs and RBPs interactions.

Taking has_circ_0033725 as an example, our circMRT predicted that it has interactions with miRNAs. According to the ENCORI database, has_circ_0033725 has interactions with 16 miRNAs (Figure 9a). circMRT predicted that has_circ_001886 has association with RBP. Databases ENCORI and Interactome^5^ shows that has_circ_001886 has interactions with AGO2, EIF4A3, FMRP, HUR, IGF2BP1, IGF2BP2, IGF2BP3, and LIN28A (Figure 9b). circMRT suggested that circRNAs has_circ_0006111, has_circ_0008173, has_circ_0012351, has_circ_0014408, has_circ_0025154, has_circ_0035174, has_circ_0080641, and has_circ_0088103 have associations with TR. According to the TRCirc database, all the above circRNAs have interactions with CTCF (Figure 9c). Moreover, has_circ_0004915 was predicted to have interactions with miRNA and RBP. From ENCORI, has_circ_0004915 has interactions with AGO2, FUS, HNRNPC, PTB, has_miR_19b-3p, has_miR_19a-3p, has_miR_2681-5p, has_miR_320c, has_miR_320b, has_miR_320d, and has_miR_4429 (Figure 9d). More details can be found in the Supplementary Material.

### Availability of Online Webserver

**FIGURE 10:**
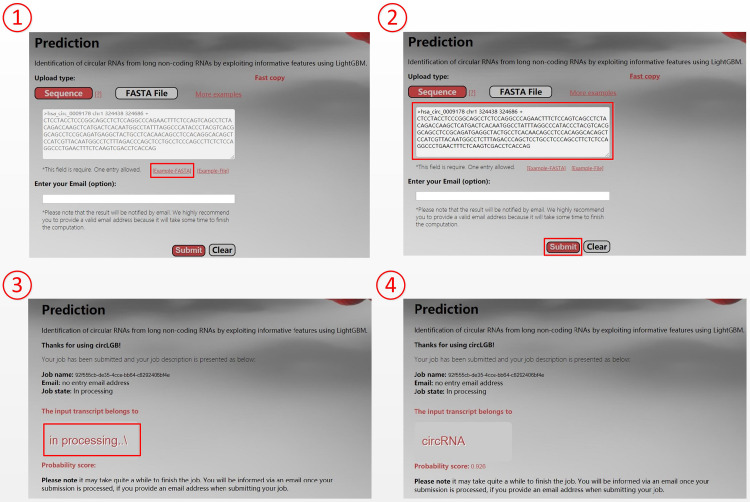
Screenshots show the prediction of hsa_circ_0009178. Users can submit query sequence in the following four steps: **(1)** Click the START button to be taken to the prediction page. **(2)** Input the hsa_circ_0009178 sequence into the input box and then click the Submit button to make prediction. **(3)** Click the Submit button to upload hsa_circ_0009178 sequence. **(4)** The web server returns the prediction result “circRNA” with a probability score of 0.926 in the Gray box.

For the convenience of researchers, we have developed an easy-to-use webserver that implements our circLGB, which is freely accessible through http://www.circlgb.com. The following description provides a step-by-step instruction on how to use the webserver to obtain the prediction result. First, users need to submit the query sequence into the input box or upload a FASTA sequence file to make a prediction. Note that the input sequence must only contain the following four canonical bases “A,” “C,” “G,” and “T.” The FASTA formatted sequence begins with a single line description, followed by lines of sequence data. The definition line is distinguished from the sequence data by a greater-than “>” character at the beginning. The rest of the definition line must contain five columns including sequence name, chromosome, start position, end position, and strand. Second, click the Submit button to upload the query sequence (FASTA file) for prediction. Upon submitting the sequence, the software will extract the features for the given sequence from a server. The Prediction page will show the job description including job ID, job name, email address, and job state. The web server will return the prediction result in the Gray box when the job is completed. Figure 10 shows an example for using the web server.

## Discussion

Here we present two machine learning-based methods, circLGB and circMRT, to classify circRNA from other lncRNAs and to predict its regulatory interactions using diverse sources of sequence-derived features, respectively. The feature section is important, in addition to the modeling approach for predicting activity. In recent years, considerable research efforts have been made in identifying circRNA, thus generating several groups of features for RNAs representation. Inspired by these studies (Pan and Xiong, 2015; Chen et al., 2018), we integrated the commonly used sequence features to generate the feature space of circLGB. To achieve optimal performance, A-to-I and A-to-I density, and IRES features were modeled in the circLGB model. The success of circLGB lies in the enriched representative features and powerful machine learning model incorporating the feature optimization strategy. Compared to existing tools, circLGB has the following merits: (i) It successfully integrates three new features that can enhance the discrimination ability for circRNA detection. (ii) It takes advantage of the feature optimization strategy to determine the most important features, thus reducing the feature dimensions and avoiding overfitting. (iii) circLGB provides a user-friendly webserver to identify circRNA for a new query RNA sequence.

Many studies focus on the interactions between circRNAs and miRNAs (e.g., TargetScan, miRanda), RBPs (e.g., ENCORI), and TRs (e.g., TRCirc). However, there is a lack of a comprehensive human circRNA regulatory information database. circMRT is an efficient computational ensemble machine learning model for simultaneous prediction of circRNA potential interacted miRNAs, RBPs, and TRs, further facilitating interpretation and its functional mechanisms. circMRT incorporates several features from other freely available web resources and toolkits, such as UCSC, TRCirc, and GraphProt. It enables the user to find the potential regulatory interactions for an unseen circRNA sequence. Together, circMRT will accelerate our efforts to understand the roles of circRNAs in biological processes related to health and disease.

Several future improvements are expected. First, we have currently designed circLGB and circMRT only for human circRNAs. They will be expanded to include other species in the future. Second, manual design of proper RNA sequence features will definitely enhance the prediction ability of models. Here, we use the commonly used sequence-derived features as well as explore three new features for RNA sequence representations and show that feature engineering really boosts the performance. Future directions can combine feature engineering and feature selection strategies for improving the prediction performance. Third, the number of the available training sample sizes have great influence on the predictive performance. However, after removing the duplications, the sample sizes of circRNA–miRNA, circRNA–RBP, and circRNA–TR interactions are relatively small, which brings a challenge for an unseen query sequence. Consequently, appropriate data augmentation techniques await exploration. Finally, though circLGB and circMRT achieve the desired performance for circRNA identification and prediction of its regulatory interactions, both of them rely heavily on the considerable domain expertise to design the feature extractor. We believe that simple and modern deep learning models will contribute to enhancements for these issues.

## Data Availability Statement

Publicly available datasets were analyzed in this study. This data can be found here: https://github.com/Peppags/circLGB-circMRT.

## Author Contributions

GZ and BY wrote the analysis source code. GZ analyzed the data and drafted the full manuscript. YD and QL collected and compiled the data from the literature and public database. ZD developed the data analysis and participated in discussion of the project. YC and XD critically revised the final manuscript. All authors contributed to the project design and read and approved the final manuscript.

## Conflict of Interest

The authors declare that the research was conducted in the absence of any commercial or financial relationships that could be construed as a potential conflict of interest.

## Footnotes

1 http://starbase.sysu.edu.cn/

2 http://licpathway.net/TRCirc/view/index

3 http://hgdownload.cse.ucsc.edu/goldenpath/hg19/phyloP46way/placentalMammals/

4 https://github.com/Microsoft/LightGBM

5 https://circinteractome.nia.nih.gov/
