# Supplementary material for: Identifying Circular RNA and Predicting Its Regulatory Interactions by Machine Learning
Source: Front Genet. 2020 Jul 21;11:655. doi: 10.3389/fgene.2020.00655 (PMC7396586; doi:10.3389/fgene.2020.00655)
Supplement: Supplementary file 6 [file Table_2.DOCX]

Supplementary Material

# Supplementary Figures and Tables

## Supplementary Figures

We assessed the performance of circLGB by introducing various transcripts from our selected data to determine the optimal sample size. We added the transcripts from our constructed data for training and testing, and evaluated the performance of circLGB on commonly used sequence-derived features. To be specific, we constructed the experimental datasets with various sample sizes from 5 thousand to 45 thousand with 5 thousand intervals for each experiment. Each dataset was randomly divided into training set and testing set with 80% and 20% classes. Figure S1 shows the ROC and PR curves of circLGB on datasets with various sample sizes under 10-time 5-fold cross-validation. Both AUC and PR-AUC values steadily increased when the sample size increased. Performance of circLGB reached the peak with 40 thousand transcripts (with AUC value of 0.976 and PR-AUC value of 0.978). Notably, both of them decreased with the number of transcripts increased. Hence, the optimum sample size was suggested to be between 40 and 45 thousand. We randomly chose 43,764 transcripts with an equal number of positive and negative data for further analysis. The constructed dataset was named as circlncRNA in this work.


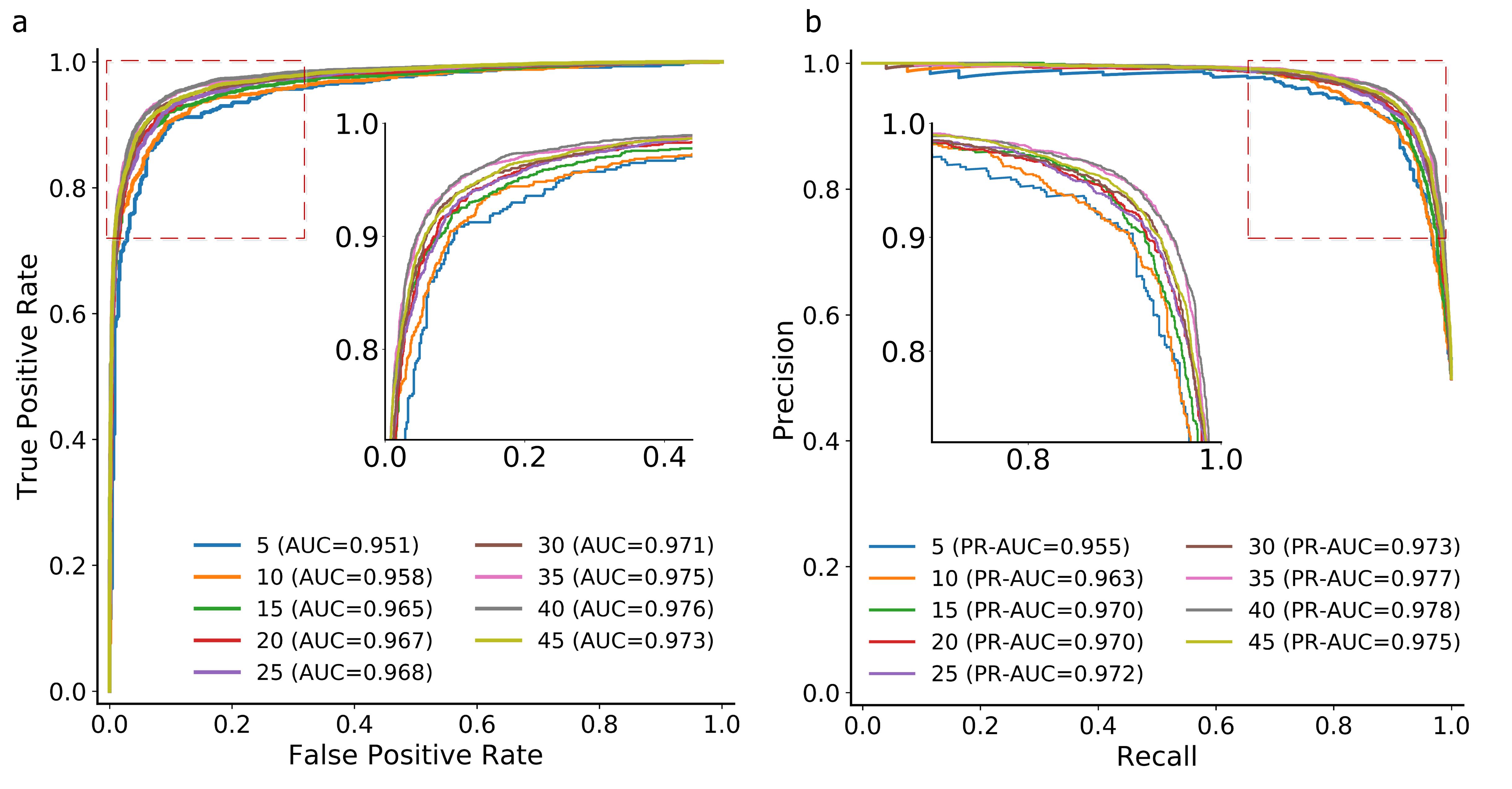


**Supplementary Figure S1.** Performance comparison of circLGB for circRNA identification with transcripts ranging from 5 to 45 thousand in interval of 5 thousand under 10-time 5-fold cross-validation. The small figures display the enlargement of the top-left corner of ROC curves and top-right corner of PR curves (marked by red dotted rectangle), respectively.


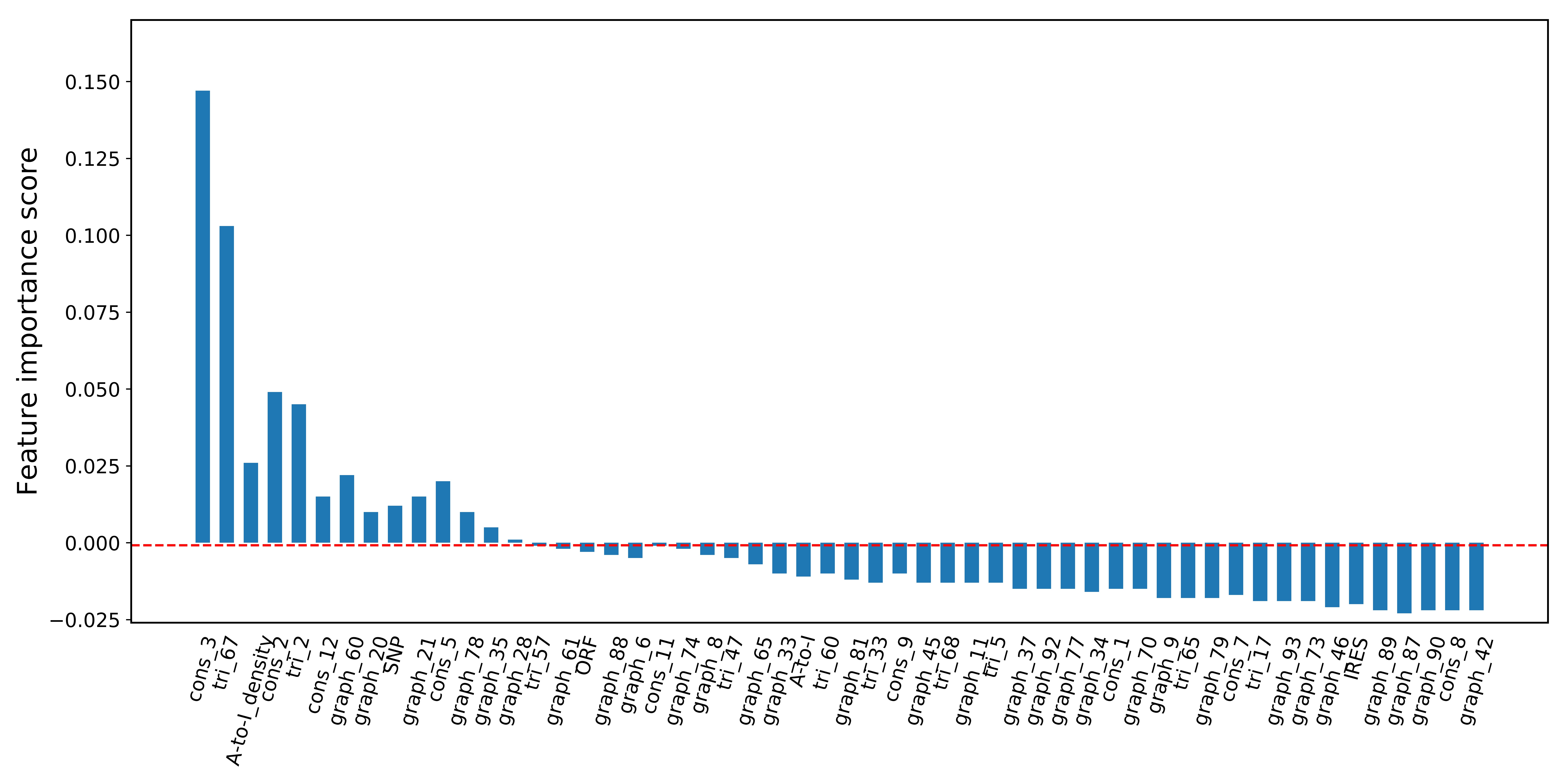


**Supplementary Figure S2.** Top 54 features from mRMR importance ranking. X-axis represents the analyzed features, cons: conservation score features; tri: sequence-based features; graph: graph features, followed by the index for each group feature. Y-axis denotes the classification importance scores. The red dotted line shows the average importance scores of the top 54 features.

## Supplementary Tables

**Supplementary Table S1.** The 191 extracted features divided into four groups for identifying circRNA from other lncRNAs.

| Feature group | Feature type | Features (#) |
| --- | --- | --- |
| Sequence composition | Length, AG, CT, CTAG, AGGT, GC content, 64 trinucleotide frequencies | 70 |
| Graph features | Top 101 graph features from GraphProt 1.0.1 | 101 |
| Conservation scores | Mean, maximum and variance of conservation score; Frequencies of bases whose conservation score is greater than 0.3, 0.6 and 0.9 as well as smaller than 0.9. Frequencies of consecutive bases whose conservation scores are greater than 0.3 | 12 |
| ATOSAAI | ALU, Tandem, ORF length, ORF propensity, | 8 |
|  | SNP density, IRES, A-to-I, |  |
| Total | - | 191 |

**Supplementary Table S2.** The 182 sequence-derived features divided into four groups for predicting circRNA regulatory interactions.

| Feature group | Feature type | Features (#) |
| --- | --- | --- |
| Sequence composition | Length, AG, CT, CTAG, AGGT, GC content, 64 trinucleotide frequencies, repeat | 71 |
| Graph features | Graph features | 101 |
| Genome context | Mean, standard deviation of conservation scores, ALU, SNP density, A-to-I, Back-splice junction, miRNA binding sites | 7 |
| Regulatory information | Methylation, transcription factors, super-enhancer | 3 |
| Total | - | 182 |

**Supplementary Table S3.** Feature importance scores by mRMR on the circlncRNA dataset.

| Rank | | Name | Score | Rank | Name | Score | Rank | Name | Score |
| --- | --- | --- | --- | --- | --- | --- | --- | --- | --- |
| 1 | | cons_3 | 0.147 | 65 | tri_29 | -0.027 | 129 | tri_53 | -0.06 |
| 2 | | tri_67 | 0.103 | 66 | graph_26 | -0.027 | 130 | tri_51 | -0.061 |
| 3 | | A-to-I density | 0.026 | 67 | graph_4 | -0.028 | 131 | graph_13 | -0.061 |
| 4 | | cons_2 | 0.049 | 68 | tri_44 | -0.028 | 132 | graph_10 | -0.062 |
| 5 | | tri_2 | 0.045 | 69 | graph_15 | -0.029 | 133 | tri_19 | -0.063 |
| 6 | | cons_12 | 0.015 | 70 | tri_9 | -0.029 | 134 | ORF_prop | -0.064 |
| 7 | | graph_60 | 0.022 | 71 | graph_2 | -0.029 | 135 | tri_45 | -0.065 |
| 8 | | graph_20 | 0.01 | 72 | graph_66 | -0.029 | 136 | graph_17 | -0.065 |
| 9 | | SNP | 0.012 | 73 | graph_75 | -0.03 | 137 | tri_30 | -0.066 |
| 10 | | graph_21 | 0.015 | 74 | cons_6 | -0.03 | 138 | tri_7 | -0.068 |
| 11 | | cons_5 | 0.02 | 75 | graph_63 | -0.03 | 139 | graph_72 | -0.07 |
| 12 | | graph_78 | 0.01 | 76 | graph_31 | -0.03 | 140 | graph_95 | -0.071 |
| 13 | | graph_35 | 0.005 | 77 | graph_41 | -0.031 | 141 | graph_100 | -0.072 |
| 14 | | graph_28 | 0.001 | 78 | tri_66 | -0.031 | 142 | tri_10 | -0.072 |
| 15 | | tri_57 | -0.001 | 79 | graph_83 | -0.031 | 143 | tri_21 | -0.073 |
| 16 | | graph_61 | -0.002 | 80 | graph_52 | -0.031 | 144 | graph_24 | -0.076 |
| 17 | | ORF | -0.003 | 81 | graph_56 | -0.032 | 145 | tri_11 | -0.077 |
| 18 | | graph_88 | -0.004 | 82 | graph_47 | -0.032 | 146 | graph_99 | -0.077 |
| 19 | | graph_6 | -0.005 | 83 | graph_64 | -0.032 | 147 | graph_76 | -0.079 |
| 20 | | cons_11 | -0.001 | 84 | graph_59 | -0.033 | 148 | tri_55 | -0.081 |
| 21 | | graph_74 | -0.002 | 85 | tri_63 | -0.032 | 149 | tri_35 | -0.082 |
| 22 | | graph_8 | -0.004 | 86 | graph_98 | -0.033 | 150 | tri_24 | -0.083 |
| 23 | | tri_47 | -0.005 | 87 | cons_4 | -0.033 | 151 | graph_43 | -0.085 |
| 24 | | graph_65 | -0.007 | 88 | graph_29 | -0.033 | 152 | tri_37 | -0.085 |
| 25 | | graph_33 | -0.01 | 89 | graph_69 | -0.033 | 153 | graph_19 | -0.088 |
| 26 | | A-to-I | -0.011 | 90 | graph_7 | -0.034 | 154 | tri_70 | -0.089 |
| 27 | | tri_60 | -0.01 | 91 | tri_59 | -0.035 | 155 | tri_6 | -0.089 |
| 28 | | graph_81 | -0.012 | 92 | graph_82 | -0.035 | 156 | tri_46 | -0.091 |
| 29 | | tri_33 | -0.013 | 93 | tri_56 | -0.035 | 157 | tri_15 | -0.093 |
| 30 | | cons_9 | -0.01 | 94 | graph_57 | -0.035 | 158 | tri_39 | -0.096 |
| 31 | | graph_45 | -0.013 | 95 | graph_101 | -0.036 | 159 | graph_71 | -0.097 |
| 32 | | tri_68 | -0.013 | 96 | tri_8 | -0.037 | 160 | graph_85 | -0.1 |
| 33 | graph_11 | | -0.013 | 97 | graph_16 | -0.037 | 161 | tri_26 | -0.101 |
| 34 | tri_5 | | -0.013 | 98 | graph_50 | -0.037 | 162 | tri_1 | -0.104 |
| 35 | graph_37 | | -0.015 | 99 | tri_58 | -0.038 | 163 | tri_41 | -0.105 |
| 36 | graph_92 | | -0.015 | 100 | graph_91 | -0.037 | 164 | graph_53 | -0.106 |
| 37 | graph_77 | | -0.015 | 101 | tri_12 | -0.038 | 165 | tri_25 | -0.109 |
| 38 | graph_34 | | -0.016 | 102 | graph_40 | -0.039 | 166 | tri_36 | -0.112 |
| 39 | cons_1 | | -0.015 | 103 | graph_38 | -0.04 | 167 | tri_20 | -0.114 |
| 40 | graph_70 | | -0.015 | 104 | tri_28 | -0.04 | 168 | graph_86 | -0.115 |
| 41 | graph_9 | | -0.018 | 105 | graph_44 | -0.041 | 169 | graph_18 | -0.117 |
| 42 | tri_65 | | -0.018 | 106 | graph_12 | -0.042 | 170 | graph_58 | -0.118 |
| 43 | graph_79 | | -0.018 | 107 | graph_1 | -0.043 | 171 | tri_38 | -0.119 |
| 44 | cons_7 | | -0.017 | 108 | graph_97 | -0.045 | 172 | tri_34 | -0.12 |
| 45 | tri_17 | | -0.019 | 109 | tri_32 | -0.046 | 173 | tri_54 | -0.121 |
| 46 | graph_93 | | -0.019 | 110 | graph_32 | -0.046 | 174 | tri_42 | -0.126 |
| 47 | graph_73 | | -0.019 | 111 | graph_96 | -0.046 | 175 | graph_67 | -0.128 |
| 48 | graph_46 | | -0.021 | 112 | ALU | -0.047 | 176 | graph_84 | -0.134 |
| 49 | IRES | | -0.02 | 113 | graph_94 | -0.047 | 177 | graph_68 | -0.135 |
| 50 | graph_89 | | -0.022 | 114 | graph_3 | -0.047 | 178 | graph_48 | -0.139 |
| 51 | graph_87 | | -0.023 | 115 | tri_50 | -0.049 | 179 | graph_39 | -0.144 |
| 52 | graph_90 | | -0.022 | 116 | graph_27 | -0.049 | 180 | tri_64 | -0.153 |
| 53 | cons_8 | | -0.022 | 117 | graph_25 | -0.049 | 181 | tri_43 | -0.155 |
| 54 | graph_42 | | -0.022 | 118 | graph_80 | -0.052 | 182 | tri_23 | -0.162 |
| 55 | graph_54 | | -0.023 | 119 | tri_31 | -0.052 | 183 | tri_22 | -0.166 |
| 56 | tri_3 | | -0.023 | 120 | graph_62 | -0.052 | 184 | tri_49 | -0.169 |
| 57 | graph_23 | | -0.023 | 121 | tri_62 | -0.053 | 185 | tri_27 | -0.173 |
| 58 | tri_48 | | -0.023 | 122 | tri_40 | -0.054 | 186 | tri_4 | -0.177 |
| 59 | graph_30 | | -0.024 | 123 | tri_18 | -0.055 | 187 | tri_13 | -0.183 |
| 60 | graph_36 | | -0.024 | 124 | graph_5 | -0.056 | 188 | tri_16 | -0.189 |
| 61 | graph_51 | | -0.025 | 125 | tri_14 | -0.056 | 189 | tri_52 | -0.197 |
| 62 | graph_55 | | -0.025 | 126 | graph_49 | -0.057 | 190 | tri_61 | -0.202 |
| 63 | graph_22 | | -0.027 | 127 | graph_14 | -0.057 | 191 | tri_69 | -0.225 |
| 64 | cons_10 | | -0.027 | 128 | Repeat | -0.057 |  |  |  |

Note: prefix cons refers to conservation features, prefix tri refers to basic sequence features, prefix graph refers to graph features, followed by the index for each group feature.

**Supplementary Table S4.** Ranked feature list of the circMI dataset.

| Rank | Name | Rank | Name | Rank | Name | Rank | Name | Rank | Name |
| --- | --- | --- | --- | --- | --- | --- | --- | --- | --- |
| 1 | ALU | 38 | graph_93 | 75 | graph_91 | 112 | graph_17 | 149 | tri_23 |
| 2 | miRNA | 39 | tri_68 | 76 | tri_3 | 113 | tri_11 | 150 | tri_44 |
| 3 | repeat | 40 | graph_36 | 77 | graph_25 | 114 | graph_19 | 151 | graph_66 |
| 4 | methylation | 41 | SNP | 78 | graph_11 | 115 | tri_69 | 152 | graph_99 |
| 5 | junction | 42 | graph_87 | 79 | tri_52 | 116 | tri_58 | 153 | graph_100 |
| 6 | cons_2 | 43 | graph_2 | 80 | graph_26 | 117 | graph_57 | 154 | tri_50 |
| 7 | cons_1 | 44 | graph_29 | 81 | tri_45 | 118 | tri_56 | 155 | tri_2 |
| 8 | graph_73 | 45 | graph_86 | 82 | graph_98 | 119 | tri_55 | 156 | graph_48 |
| 9 | graph_6 | 46 | tri_1 | 83 | graph_92 | 120 | tri_42 | 157 | tri_9 |
| 10 | TF | 47 | graph_60 | 84 | tri_63 | 121 | graph_82 | 158 | graph_51 |
| 11 | H3k27ac | 48 | graph_23 | 85 | tri_31 | 122 | tri_35 | 159 | tri_5 |
| 12 | graph_56 | 49 | tri_64 | 86 | tri_54 | 123 | tri_32 | 160 | tri_12 |
| 13 | tri_39 | 50 | graph_81 | 87 | tri_43 | 124 | tri_61 | 161 | graph_18 |
| 14 | graph_44 | 51 | graph_15 | 88 | tri_51 | 125 | tri_26 | 162 | tri_10 |
| 15 | graph_83 | 52 | tri_21 | 89 | graph_69 | 126 | tri_24 | 163 | tri_59 |
| 16 | graph_58 | 53 | graph_76 | 90 | graph_67 | 127 | graph_90 | 164 | graph_70 |
| 17 | graph_79 | 54 | graph_38 | 91 | graph_80 | 128 | graph_55 | 165 | tri_48 |
| 18 | tri_40 | 55 | graph_89 | 92 | graph_78 | 129 | graph_16 | 166 | graph_5 |
| 19 | graph_97 | 56 | graph_30 | 93 | graph_42 | 130 | graph_50 | 167 | tri_28 |
| 20 | tri_41 | 57 | graph_43 | 94 | graph_68 | 131 | graph_47 | 168 | tri_57 |
| 21 | tri_17 | 58 | graph_71 | 95 | graph_40 | 132 | graph_1 | 169 | tri_66 |
| 22 | graph_61 | 59 | graph_84 | 96 | graph_27 | 133 | tri_47 | 170 | tri_53 |
| 23 | graph_31 | 60 | graph_4 | 97 | graph_59 | 134 | graph_8 | 171 | graph_62 |
| 24 | graph_20 | 61 | tri_20 | 98 | graph_35 | 135 | graph_94 | 172 | graph_64 |
| 25 | graph_74 | 62 | tri_8 | 99 | graph_53 | 136 | graph_13 | 173 | graph_88 |
| 26 | graph_22 | 63 | graph_52 | 100 | graph_49 | 137 | tri_34 | 174 | tri_15 |
| 27 | tri_6 | 64 | tri_62 | 101 | graph_46 | 138 | graph_41 | 175 | graph_45 |
| 28 | graph_65 | 65 | tri_38 | 102 | graph_63 | 139 | tri_65 | 176 | tri_30 |
| 29 | graph_3 | 66 | tri_60 | 103 | graph_21 | 140 | graph_72 | 177 | tri_29 |
| 30 | A-to-I | 67 | graph_28 | 104 | graph_24 | 141 | tri_19 | 178 | tri_46 |
| 31 | tri_27 | 68 | graph_12 | 105 | tri_16 | 142 | graph_37 | 179 | graph_101 |
| 32 | graph_7 | 69 | graph_34 | 106 | tri_33 | 143 | graph_39 | 180 | tri_22 |
| 33 | graph_9 | 70 | graph_77 | 107 | tri_7 | 144 | tri_25 | 181 | tri_67 |
| 34 | graph_54 | 71 | tri_4 | 108 | tri_36 | 145 | graph_85 | 182 | tri_70 |
| 35 | graph_33 | 72 | graph_32 | 109 | graph_10 | 146 | tri_37 |  |  |
| 36 | tri_49 | 73 | graph_75 | 110 | tri_18 | 147 | graph_14 |  |  |
| 37 | tri_13 | 74 | graph_95 | 111 | tri_14 | 148 | graph_96 |  |  |

**Supplementary Table S5.** Ranked feature list of the circRBP dataset.

| Rank | Name | Rank | Name | Rank | Name | Rank | Name | Rank | | Name | |
| --- | --- | --- | --- | --- | --- | --- | --- | --- | --- | --- | --- |
| 1 | junction | 38 | graph_77 | 75 | graph_64 | 112 | graph_69 | 149 | | graph_48 | |
| 2 | repeat | 39 | graph_43 | 76 | graph_78 | 113 | tri_16 | 150 | | tri_60 | |
| 3 | cons_2 | 40 | graph_38 | 77 | graph_2 | 114 | tri_38 | 151 | | graph_8 | |
| 4 | ALU | 41 | tri_37 | 78 | miRNA | 115 | graph_98 | 152 | | tri_45 | |
| 5 | methylation | 42 | graph_27 | 79 | tri_49 | 116 | graph_29 | 153 | | tri_24 | |
| 6 | cons_1 | 43 | graph_4 | 80 | graph_62 | 117 | tri_9 | 154 | | tri_15 | |
| 7 | H3k27ac | 44 | tri_40 | 81 | graph_83 | 118 | graph_95 | 155 | | tri_63 | |
| 8 | TF | 45 | tri_68 | 82 | graph_99 | 119 | graph_75 | 156 | | tri_59 | |
| 9 | graph_28 | 46 | graph_61 | 83 | graph_31 | 120 | tri_7 | 157 | | tri_46 | |
| 10 | graph_33 | 47 | tri_36 | 84 | tri_5 | 121 | graph_100 | 158 | | tri_69 | |
| 11 | A-to-I | 48 | graph_82 | 85 | tri_42 | 122 | graph_12 | 159 | | tri_44 | |
| 12 | graph_59 | 49 | graph_93 | 86 | graph_3 | 123 | graph_34 | 160 | | tri_64 | |
| 13 | graph_16 | 50 | tri_14 | 87 | tri_17 | 124 | tri_22 | 161 | | graph_5 | |
| 14 | graph_22 | 51 | graph_50 | 88 | tri_55 | 125 | graph_14 | 162 | | graph_66 | |
| 15 | graph_7 | 52 | graph_55 | 89 | tri_11 | 126 | tri_54 | 163 | | tri_66 | |
| 16 | graph_81 | 53 | tri_3 | 90 | graph_40 | 127 | graph_80 | 164 | | tri_31 | |
| 17 | graph_101 | 54 | graph_9 | 91 | tri_43 | 128 | tri_27 | 165 | | tri_6 | |
| 18 | graph_88 | 55 | tri_13 | 92 | tri_20 | 129 | tri_50 | 166 | | tri_29 | |
| 19 | SNP | 56 | tri_39 | 93 | graph_37 | 130 | graph_91 | 167 | | tri_67 | |
| 20 | graph_19 | 57 | graph_25 | 94 | tri_35 | 131 | tri_10 | 168 | | tri_12 | |
| 21 | graph_51 | 58 | graph_56 | 95 | graph_65 | 132 | graph_86 | 169 | | graph_85 | |
| 22 | graph_30 | 59 | graph_68 | 96 | graph_47 | 133 | tri_25 | 170 | | tri_34 | |
| 23 | graph_35 | 60 | graph_79 | 97 | graph_49 | 134 | tri_56 | 171 | | tri_23 | |
| 24 | graph_6 | 61 | graph_53 | 98 | tri_52 | 135 | tri_57 | 172 | | graph_92 | |
| 25 | graph_96 | 62 | graph_17 | 99 | graph_10 | 136 | tri_62 | 173 | | graph_21 | |
| 26 | graph_97 | 63 | graph_89 | 100 | tri_26 | 137 | tri_21 | 174 | | tri_18 | |
| 27 | graph_74 | 64 | tri_65 | 101 | graph_15 | 138 | tri_1 | 175 | | tri_19 | |
| 28 | graph_45 | 65 | graph_94 | 102 | tri_2 | 139 | tri_41 | 176 | | graph_58 | |
| 29 | graph_87 | 66 | graph_20 | 103 | tri_47 | 140 | graph_39 | 177 | | tri_61 | |
| 30 | graph_23 | 67 | tri_33 | 104 | graph_54 | 141 | graph_13 | 178 | | graph_60 | |
| 31 | graph_32 | 68 | graph_1 | 105 | graph_52 | 142 | tri_30 | 179 | | tri_28 |  |
| 32 | graph_71 | 69 | graph_44 | 106 | tri_58 | 143 | graph_46 | 180 | | tri_48 |  |
| 33 | graph_36 | 70 | graph_90 | 107 | graph_76 | 144 | tri_32 | 181 | | graph_70 |  |
| 34 | graph_72 | 71 | graph_73 | 108 | graph_11 | 145 | tri_51 | 182 | | tri_70 |  |
| 35 | graph_63 | 72 | tri_4 | 109 | graph_84 | 146 | tri_8 |  | |  |  |
| 36 | graph_24 | 73 | graph_26 | 110 | graph_41 | 147 | tri_53 |  |  | |  |
| 37 | graph_18 | 74 | graph_57 | 111 | graph_67 | 148 | graph_42 |  |  | |  |

**Supplementary Table S6.** Ranked feature list of the circTR dataset.

| Rank | Name | Rank | Name | Rank | Name | Rank | Name | Rank | Name | |  |
| --- | --- | --- | --- | --- | --- | --- | --- | --- | --- | --- | --- |
| 1 | junction | 38 | graph_57 | 75 | graph_72 | 112 | tri_31 | 149 | tri_44 | |  |
| 2 | methylation | 39 | graph_50 | 76 | graph_19 | 113 | graph_62 | 150 | tri_22 | |  |
| 3 | cons_2 | 40 | graph_64 | 77 | graph_83 | 114 | graph_41 | 151 | tri_23 | |  |
| 4 | repeat | 41 | graph_40 | 78 | graph_20 | 115 | tri_3 | 152 | tri_14 | |  |
| 5 | H3k27ac | 42 | tri_42 | 79 | graph_15 | 116 | tri_50 | 153 | graph_76 | |  |
| 6 | ALU | 43 | graph_45 | 80 | graph_11 | 117 | graph_13 | 154 | tri_18 | |  |
| 7 | cons_1 | 44 | graph_32 | 81 | tri_26 | 118 | tri_41 | 155 | tri_45 | |  |
| 8 | TF | 45 | tri_37 | 82 | tri_27 | 119 | graph_69 | 156 | tri_21 | |  |
| 9 | graph_74 | 46 | tri_36 | 83 | tri_2 | 120 | tri_19 | 157 | graph_85 | |  |
| 10 | graph_9 | 47 | tri_16 | 84 | tri_52 | 121 | tri_56 | 158 | tri_34 | |  |
| 11 | graph_56 | 48 | graph_47 | 85 | graph_94 | 122 | tri_33 | 159 | tri_5 | |  |
| 12 | graph_55 | 49 | graph_27 | 86 | graph_67 | 123 | graph_80 | 160 | graph_60 | |  |
| 13 | graph_33 | 50 | graph_79 | 87 | tri_55 | 124 | tri_11 | 161 | tri_46 | |  |
| 14 | graph_59 | 51 | graph_87 | 88 | graph_34 | 125 | tri_35 | 162 | tri_53 | |  |
| 15 | SNP | 52 | tri_58 | 89 | graph_49 | 126 | tri_7 | 163 | tri_61 | |  |
| 16 | graph_101 | 53 | graph_14 | 90 | graph_53 | 127 | tri_8 | 164 | tri_10 | |  |
| 17 | A-to-I | 54 | graph_30 | 91 | graph_29 | 128 | graph_25 | 165 | tri_64 | |  |
| 18 | graph_35 | 55 | graph_18 | 92 | graph_75 | 129 | graph_52 | 166 | graph_66 | |  |
| 19 | graph_28 | 56 | tri_65 | 93 | graph_6 | 130 | graph_39 | 167 | tri_47 | |  |
| 20 | graph_51 | 57 | graph_38 | 94 | graph_84 | 131 | graph_65 | 168 | graph_8 | |  |
| 21 | graph_88 | 58 | graph_91 | 95 | tri_49 | 132 | graph_42 | 169 | tri_57 | |  |
| 22 | graph_89 | 59 | tri_68 | 96 | graph_3 | 133 | graph_61 | 170 | graph_21 | |  |
| 23 | tri_39 | 60 | graph_37 | 97 | graph_68 | 134 | tri_67 | 171 | tri_24 | |  |
| 24 | graph_17 | 61 | graph_26 | 98 | tri_13 | 135 | tri_32 | 172 | tri_66 | |  |
| 25 | graph_63 | 62 | graph_24 | 99 | graph_36 | 136 | tri_51 | 173 | tri_63 | |  |
| 26 | graph_82 | 63 | graph_54 | 100 | tri_9 | 137 | tri_12 | 174 | tri_69 | |  |
| 27 | graph_81 | 64 | graph_93 | 101 | graph_31 | 138 | graph_100 | 175 | tri_48 | |  |
| 28 | graph_97 | 65 | graph_10 | 102 | tri_4 | 139 | graph_58 | 176 | miRNA | |  |
| 29 | graph_77 | 66 | graph_98 | 103 | tri_15 | 140 | tri_17 | 177 | graph_5 | |  |
| 30 | graph_23 | 67 | graph_4 | 104 | graph_46 | 141 | tri_6 | 178 | tri_62 | |  |
| 31 | graph_90 | 68 | graph_7 | 105 | graph_43 | 142 | tri_20 | 179 | graph_92 | |  |
| 32 | graph_22 | 69 | graph_1 | 106 | graph_86 | 143 | graph_48 | 180 | graph_70 | |  |
| 33 | graph_2 | 70 | tri_25 | 107 | tri_1 | 144 | graph_99 | 181 | tri_28 | |  |
| 34 | graph_12 | 71 | tri_40 | 108 | graph_71 | 145 | tri_59 | 182 | tri_70 | |  |
| 35 | graph_16 | 72 | graph_44 | 109 | tri_30 | 146 | tri_60 |  |  | |  |
| 36 | graph_78 | 73 | graph_95 | 110 | tri_54 | 147 | tri_38 |  |  |  |  |
| 37 | graph_96 | 74 | graph_73 | 111 | tri_43 | 148 | tri_29 |  |  | | |
